# Profilin and Non-Canonical Wnt Signaling: Coordinating Cytoskeletal Dynamics from Development to Disease

**DOI:** 10.3390/jdb13030031

**Published:** 2025-09-01

**Authors:** Samira Alam, Danielle Duncan, Sharmin Hasan

**Affiliations:** Department of Biological Sciences, Sam Houston State University, Huntsville, TX 77341, USA; sxa238@shsu.edu (S.A.); dxd117@shsu.edu (D.D.)

**Keywords:** Profilin, non-canonical Wnt, signaling, actin cytoskeleton, isoform, gene expression, gene function

## Abstract

Vertebrate embryonic development relies on tightly regulated signaling pathways that guide morphogenesis, cell fate specification, and tissue organization. Among these, the Wnt signaling pathway plays a central role, orchestrating key developmental events. The non-canonical Wnt pathways, including the Planar Cell Polarity and Wnt/Ca^2+^ branches, are especially critical for regulating cytoskeletal dynamics during gastrulation. Recent studies highlight that these pathways interface with cytoskeletal effectors to control actin remodeling in response to extracellular cues. One such effector is Profilin, a small, evolutionarily conserved actin-binding protein that modulates actin polymerization and cellular architecture. Profilins, particularly Profilin1 and 2, are known to interact with Daam1, a formin protein downstream of PCP signaling, thereby linking Wnt signals to actin cytoskeletal regulation. Emerging evidence suggests that Profilins are active signaling intermediates that contribute to morphogenetic processes. Their context-dependent interactions and differential expression across species also suggest that they play specialized roles in development and disease. This review synthesizes the current understanding of Profilin’s role in non-canonical Wnt signaling, examining its molecular interactions and contributions to cytoskeletal control during development. By integrating data across model systems, we aim to clarify how Profilins function at the intersection of signaling and cytoskeletal dynamics, with implications for both developmental biology and disease pathogenesis.

## 1. Introduction

Vertebrate embryonic development is a critical phase, during which organs and body systems are progressively established. This intricate process is orchestrated by the coordinated interplay of multiple signaling pathways, with the Wnt signaling pathway playing a pivotal role in ensuring proper embryogenesis [1]. The term “Wnt” derives from a fusion of *wingless*, a gene first identified in *Drosophila melanogaster*, and *Int-1*, a proto-oncogene discovered in mice [2,3]. These genes share functional and positional similarities during morphogenesis [4].

Wnt proteins are a large family of secreted glycoproteins that function as key signaling molecules, regulating a diverse array of biological processes during development and in adult tissue homeostasis [5]. This evolutionarily conserved pathway, potentially predating the emergence of multicellular organisms, has undergone significant expansion of its gene family. For instance, humans and mice possess 19 Wnt genes, *Drosophila* has 7, *C. elegans* has 5 [5], and zebrafish possess up to 25 due to whole-genome duplication [6,7,8].

In vertebrate development, Wnt signaling regulates a wide array of processes, including axis formation, tissue homeostasis, cell proliferation, motility, and organogenesis, such as head development and bone formation [5,9,10,11,12]. It also plays crucial roles in limb initiation [13], lens development [14], and hematopoietic stem cell fate [15]. During gastrulation, Wnt signaling, particularly through the non-canonical Planar Cell Polarity (PCP) pathway, regulates convergent extension movements critical for axial elongation, involving effectors such as Daam1 and Profilins [16,17,18,19].

Beyond embryogenesis, Wnt signaling is essential for adult tissue maintenance and regeneration. It regulates stem cell populations in the mammary gland [20], intestinal epithelium [21], skin [22], heart [23], and liver [24] and is essential for neural crest specification and the differentiation of pigment cells [25]; it also participates in liver regeneration [26]. Dysregulation of Wnt signaling is implicated in neurodegenerative diseases such as Alzheimer’s [27] and Parkinson’s disease [28]. Aberrant Wnt signaling contributes to cancers of the colon [29], breast [30], lung [31], and skin [12].

Wnt proteins undergo lipid modification, notably the covalent attachment of palmitoleic acid, which enhances their hydrophobicity, promotes receptor interaction, and enables their function as morphogens [32]. These modifications, along with tightly regulated intracellular trafficking and secretion, are essential for their signaling potency. Wnt signaling begins when Wnt ligands bind to the cysteine-rich extracellular domain of Frizzled (Fz) receptors [33,34]. The number of Fz genes varies by species, as mammals possess 10 [35,36], while zebrafish are predicted to have at least 17 [8]. Canonical Wnt signaling also requires co-receptors such as LRP5/6, which are phosphorylated at conserved motifs to inhibit GSK3β, a key regulatory kinase [37,38,39]. Wnt ligands exhibit receptor specificity, with different classes preferentially binding to distinct extracellular β-propeller-EGF domains of LRP5/6 [40].

Wnt signal transduction continues intracellularly via Dishevelled (Dvl in mammals, Dsh in *Drosophila*) [41]. Wnt ligands are often categorized by their pathway preference; notable canonical (β-catenin-dependent) ligands include Wnt1, Wnt2, Wnt3a, and Wnt8, whereas non-canonical (β-catenin-independent) ligands include Wnt4, Wnt5a/b, Wnt6, Wnt7a, and Wnt11 [9]. However, recent evidence demonstrates that this distinction is not rigid. Wnt signaling outcomes are highly context-dependent, shaped by receptor/co-receptor expression profiles [42]. For example, canonical ligand Wnt3a can signal through PKC in a non-canonical pathway [43], and non-canonical Wnt5a and Wnt11 can activate β-catenin under certain conditions [44,45,46]. These findings highlight the remarkable versatility and plasticity of Wnt signaling.

Beyond transcriptional control, recent studies emphasize the role of Wnt signaling in modulating cytoskeletal dynamics, particularly through non-canonical branches such as the PCP and Wnt/Ca^2+^ pathways. These pathways regulate actin remodeling to guide cell polarity, motility, and morphogenesis. Among the critical cytoskeletal effectors downstream of these pathways are Profilin family proteins, small, highly conserved actin-binding proteins that facilitate actin filament turnover in response to external cues [47]. Profilin was first isolated from calf spleens as part of an actin complex [48] and is primarily associated with β- and γ-actin isoforms. It plays a central role in actin polymerization and cytoskeletal reorganization [49]. Notably, Profilin interacts with Daam1, a key PCP effector, linking Wnt signals to precise spatial control of the actin cytoskeleton [17]. Given their pivotal role in orchestrating cytoskeletal dynamics during gastrulation and other morphogenetic events, Profilins are emerging as essential integrators of Wnt signaling. In this review, we explore the multifaceted functions of Profilin in non-canonical Wnt pathways; its molecular interactions, expression, and functional conservation across species; and its broader roles in development and disease.

## 2. Canonical or Wnt/β-Catenin-Dependent Pathway

**Figure 1:**
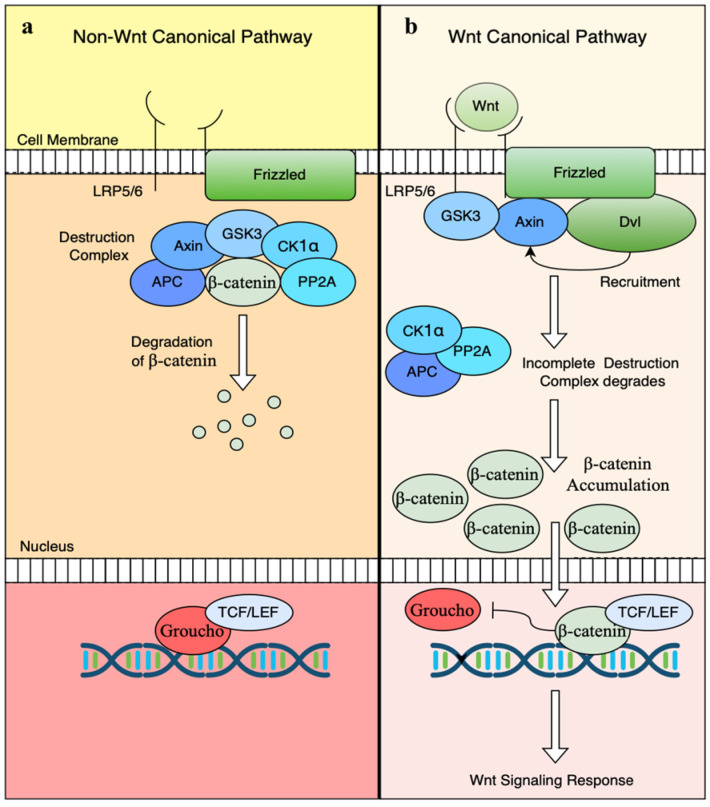
Canonical Wnt/β-catenin signaling with and without Wnt ligand. (**a**) In the absence of Wnt, the destruction complex degrades β-catenin, preventing nuclear signaling. (**b**) When Wnt ligands bind, the destruction complex is disrupted, allowing β-catenin to accumulate and enter the nucleus. Nuclear β-catenin displaces Groucho from TCF/LEF transcription factors, activating Wnt target gene expression.

Upon receptor engagement, the Wnt signal diverges into two principal branches, the canonical (β-catenin-dependent) pathway and the non-canonical (β-catenin-independent) pathway, each orchestrating distinct yet often complementary cellular outcomes. In the absence of Wnt ligands (basal state), the Frizzled (Fz) receptors and the low-density lipoprotein receptor-related protein 5/6 (LRP5/6) co-receptors remain inactive, and a cytoplasmic destruction complex phosphorylates β-catenin, targeting it for ubiquitination and proteasomal degradation (Figure 1a) [9]. This destruction complex is composed of Glycogen Synthase Kinase 3 (GSK3), Casein Kinase 1 (CK1), Axin, Adenomatous Polyposis Coli (APC), β-TrCP (an E3 ubiquitin ligase), and Protein Phosphatase 2A (PP2A) [50,51,52,53,54].

Upon Wnt ligand binding (activated state), the signal is initiated when Wnt ligands bind to Fz receptors and LRP5/6 co-receptors [5,37,50]. Ligand binding induces the recruitment of Dishevelled (Dvl) to the plasma membrane, leading to phosphorylation of LRP5/6 by GSK3β and CK1α. Phosphorylated LRP5/6 then recruits Axin to the membrane, disrupting the destruction complex. As a result, β-catenin is stabilized, accumulates in the cytoplasm, and translocates to the nucleus, where it displaces Groucho from TCF/LEF transcription factors to activate target gene expression (Figure 1b) [55,56]. Nuclear β-catenin recruits co-activators such as CBP/p300, Pygo, BCL9, and BRG1, thereby initiating the transcription of target genes [57]. Notably, in early vertebrate development, TCF/LEF drives the expression of the homeobox genes Siamois and Twin. These genes encode proteins that establish the Spemann–Mangold Organizer, known as the shield in zebrafish, which defines the future dorsal side of the embryo and patterns the embryonic axis [58].

Wnt signaling is tightly regulated not only by its core components but also by a complex network of extracellular and membrane-associated modulators that influence ligand availability, receptor activation, and downstream signal transduction. Among the secreted antagonists, secreted Frizzled-related proteins (sFRPs) and Wnt inhibitory factor-1 (WIF-1) function as decoy receptors that sequester Wnt ligands, thereby preventing their interaction with Frizzled (Fz) receptors. sFRPs contain a C-terminal netrin-like domain that mediates Wnt binding, whereas WIF-1 harbors a conserved WIF domain conferring high-affinity interaction with Wnt proteins [59,60]. Notably, sFRPs can paradoxically potentiate Wnt/β-catenin signaling in certain cellular contexts, a concentration- and receptor profile-dependent phenomenon [61]. Sclerostin (SOST) is another extracellular Wnt inhibitor that binds to the LRP5/6 co-receptors and blocks canonical Wnt pathway activation [62]. Additionally, Wnt ligands can be enzymatically inactivated by Tiki, a metalloprotease that cleaves Wnt proteins, and Notum, a deacylase that removes essential palmitoleic acid modifications, thereby preventing Wnt-receptor interactions [63,64].

Beyond ligand inactivation and decoy mechanisms, several modulators regulate the assembly and stability of the Wnt receptor complex. The Dickkopf (DKK) family proteins, including DKK1, DKK3, and DKK4, inhibit canonical Wnt signaling by binding to LRP5/6 and recruiting the co-receptors Kremen1 and Kremen2, leading to LRP internalization and disruption of the Wnt-Fz complex [65]. DKK1 also participates in a negative feedback loop, as it is a transcriptional target of β-catenin/TCF [66,67]. In contrast, R-spondins are positive regulators that potentiate Wnt signaling by binding to leucine-rich repeat-containing G protein-coupled receptors (LGR4/5/6) [68]. R-spondins simultaneously interact with the transmembrane E3 ubiquitin ligases RNF43 and ZNRF3, which normally ubiquitinate Fz receptors to promote their internalization and degradation [69,70]. The R-spondin–LGR complex induces auto-ubiquitination and removal of RNF43/ZNRF3 from the membrane, thereby stabilizing Fz receptors and enhancing signal transduction [71,72,73,74]. R-spondins can also counteract DKK1-mediated inhibition by disrupting the DKK1–Kremen–LRP6 complex, thus preserving LRP6 availability for ligand engagement [75]. Norrin, although not a member of the Wnt protein family, mimics Wnt activity through structural loop similarity and binds to Fz4 and LRP5/6 to activate canonical Wnt signaling, even in low-Wnt environments [76].

Context-specific receptor modules further diversify Wnt pathway regulation. For example, Wnt4 and Wnt11 bind to the cysteine-rich domain (CRD) of the muscle-specific receptor tyrosine kinase (MuSK), activating the β-catenin pathway to promote acetylcholine receptor (AChR) clustering and neuromuscular junction (NMJ) differentiation [77]. This occurs through increased cytoplasmic and nuclear β-catenin levels, accompanied by reduced β-catenin phosphorylation. Interestingly, Wnt4 and Wnt11 contribute to NMJ formation by activating both the canonical Wnt/β-catenin and the Vangl2-dependent non-canonical Planar Cell Polarity (PCP) signaling pathways [77]. Likewise, Gpr124, a G protein-coupled receptor, and RECK, a GPI-anchored glycoprotein, form a Wnt7-specific signaling module. Reck binds to Wnt7 and associates with Gpr124 to form a ternary complex with Fz and LRP5/6, activating canonical Wnt/β-catenin signaling critical for CNS vascularization and axon tract development [78,79,80]. Together, these extracellular inhibitors, co-receptors, enzymatic modifiers, and context-dependent agonists orchestrate precise spatial and temporal control of Wnt/β-catenin signaling, which is essential for body axis formation, mesoderm induction, and numerous tissue-specific developmental processes [81].

## 3. Non-Canonical Wnt Signaling Pathway/β-Catenin-Independent Pathway

The non-canonical Wnt pathway, also referred to as the β-catenin-independent pathway, regulates a wide range of essential developmental processes, is indispensable for vertebrate development, and drives gastrulation movements, neural tube closure, and organogenesis [82,83]. In contrast to the canonical pathway, non-canonical signaling does not primarily control cell fate decisions but rather orchestrates the morphogenetic movements required for proper embryogenesis [9].

This pathway encompasses a diverse array of molecular components, reflecting its mechanistic complexity. Key players include Van Gogh-like (Vangl) proteins, Cadherin EGF LAG Seven-pass G-type Receptors (Celsr), Prickle, Missing in Metastasis (MIM), and Profilin1, among others [17,84,85,86,87]. Genetic ablation of any of the core components illustrates the pathway’s critical developmental roles. For example, loss of Vangl (also known as Trilobite/Strabismus) or Celsr (also called Flamingo) results in aberrant Planar Cell Polarity (PCP) phenotypes in Drosophila, such as swirling patterns of wing hairs observed in Dishevelled (Dsh) mutants [86]. Similarly, Vangl mutations disrupt ommatidial organization in the Drosophila eye, while Celsr1 mutations in mice lead to misoriented cochlear hair cells and neural tube defects [85]. Furthermore, MIM depletion in Xenopus embryos results in anterior neural tube closure defects [87]. In human embryonic stem cells, non-canonical ligands promote mesodermal differentiation and enhance hematopoietic cell production [15]. Additionally, this pathway is vital for lens development via the Wnt/JNK axis. Disruption of this signaling is linked to congenital cataracts, open-angle glaucoma, and microphthalmia [88,89]. Beyond embryogenesis, non-canonical Wnt signaling regulates tissue homeostasis and exhibits tumor-suppressive functions in select contexts. For example, in hematopoietic tissues, Wnt5a inhibits B-cell proliferation and acts as a tumor suppressor, with pathway dysregulation implicated in the development of leukemia [90,91]. Conversely, aberrant non-canonical Wnt activity contributes to the pathogenesis of several solid tumors, including pancreatic [92], lung [93], breast [94], and gastric cancers [95]. Mechanistically, β-catenin-independent Wnt signaling is broadly classified into two principal branches: the Wnt/Ca^2+^ pathway and the Planar Cell Polarity (PCP) pathway. Each branch elicits distinct intracellular responses, enabling precise and dynamic control of cell behavior across a wide range of developmental and pathological contexts.

### 3.1. Profilin Function in the Non-Canonical Wnt/Ca^2+^ Pathway

The non-canonical Wnt/Ca^2+^ signaling pathway represents a distinct arm of Wnt signaling, mechanistically separate from the canonical (β-catenin-dependent) and Planar Cell Polarity (PCP) branches. It plays a critical role in modulating intracellular calcium levels and activating calcium-sensitive signaling cascades. Profilin1 (PFN1) has emerged as an important mediator of cell migration and adhesion, potentially linking integrin signaling to the activation of the Wnt/Ca^2+^ pathway [96].

**Figure 2:**
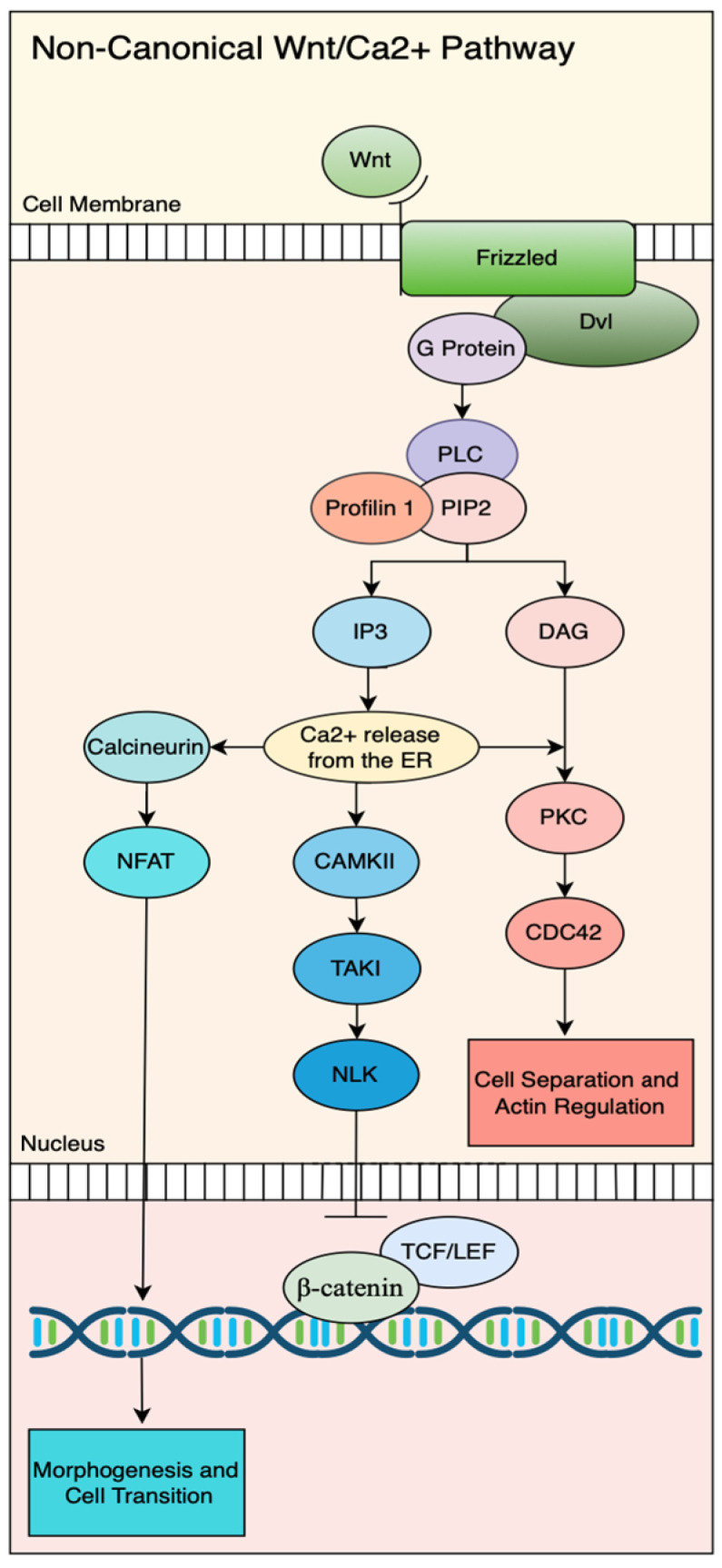
The non-canonical Wnt/Ca^2+^ signaling pathway. Wnt ligand binding to the Frizzled receptor activates G proteins and Dvl, triggering PLC to produce IP3 and DAG from PIP2. Profilin1 can interact with phosphoinositide lipids, PIP2/IP3. IP3 induces Ca^2+^ release from the endoplasmic reticulum, activating multiple downstream effectors, including Calcineurin/NFAT and CAMKII/TAK1/NLK pathways, while DAG activates PKC/CDC42 signaling. These pathways ultimately regulate distinct cellular processes, including morphogenesis and cell transition.

Activation of the Wnt/Ca^2+^ pathway begins when the Wnt ligands bind to Frizzled (Fz) receptors, leading to the recruitment of the intracellular scaffold protein Dishevelled (Dvl) in concert with heterotrimeric G proteins. This complex activates phospholipase Cδ4a (Plcδ4a), which hydrolyzes phosphatidylinositol 4,5-bisphosphate (PIP2) into the secondary messengers diacylglycerol (DAG) and inositol 1,4,5-trisphosphate (IP3) [97]. IP3 binds to its receptors InsP3Rs on the endoplasmic reticulum, triggering the release of Ca^2+^ into the cytoplasm. The resulting calcium surge activates several calcium-dependent effectors, including calcineurin, calmodulin-dependent kinase II (CaMKII) [98], and protein kinase C (PKC) [99], the latter also being activated by DAG [91]. PKC activation, in turn, stimulates the small Rho GTPase CDC42, which regulates cell polarity, actomyosin contractility, and convergent extension movements [100]. PFN1 plays a crucial role in the Wnt/Ca^2+^ pathway by binding with phosphoinositide lipids, particularly PIP2 and PIP3, and modulating downstream calcium-dependent signaling [101,102] (Figure 2). Profilin high-affinity binding to PIP2 forms stable complexes in pure PIP2 micelles. By sequestering PIP2 molecules, Profilin directly competes with PLC for substrate access, effectively inhibiting PIP2 hydrolysis and dampening the downstream calcium signaling cascade by limiting IP3 production and subsequent ER calcium release [102]. This competitive inhibition creates a negative regulatory mechanism that raises the activation threshold for Wnt/Ca^2+^ signaling, ensuring that calcium responses occur only under appropriate cellular conditions. However, this inhibitory effect can be overcome when PLC-γ1 becomes phosphorylated by receptor tyrosine kinases, which increases PLC’s affinity for PIP2 and allows it to outcompete Profilin [103]. A recent study reported Profilin1 as a key regulator of PI(4,5)P_2_, the most abundant PPI in cells. Under epidermal growth factor (EGF) stimulation, Profilin1 depletion does not affect PI(4,5)P_2_ hydrolysis but significantly enhances plasma membrane (PM) accumulation of PI3K-generated PPIs, including PI(3,4,5)P_3_ and PI(3,4)P_2_ [104].

PFN1 also binds to proline-rich ligands, highlighting its role in actin cytoskeletal dynamics and cellular adhesion [105,106]. In the context of bladder cancer, PFN1 has been identified as a critical regulator of non-canonical Wnt/Ca^2+^ signaling. Muscle-invasive bladder cancers exhibit reduced PFN1 expression, which correlates with decreased levels of PLCβ4 and suppression of this signaling axis [96,107]. In metastatic bladder cancer cells, PFN1 facilitates integrin-mediated activation of the Wnt/Ca^2+^ pathway via PIP2, promoting fibronectin adhesion, actin remodeling, and cell motility. Silencing PFN1 leads to a marked reduction in PLCβ2/4 levels and impairs these cellular functions, emphasizing its role as a modulator of calcium-dependent signaling. Notably, tumor xenografts derived from PFN1-silenced cells in mice formed significantly smaller, non-metastatic tumors [96]. These findings contrast with those in breast cancer, where PFN1 downregulation enhances motility [105], underscoring the context-dependent nature of Profilin’s role in cancer [108].

Despite growing evidence linking PFN1 to the Wnt/Ca^2+^ signaling axis, several fundamental questions remain. Future studies should focus on dissecting the precise molecular mechanisms by which PFN1 regulates the recruitment and activity of PLC isoforms in different tissue contexts. The temporal dynamics of PFN1 interactions with phosphoinositides and calcium-sensitive effectors during developmental and pathological processes also warrant further investigation. Moreover, the development of in vivo models with tissue-specific PFN1 deletions will be instrumental in determining its role in morphogenesis and cancer metastasis. Additionally, proteomic approaches may help uncover additional PFN1-interacting partners within the Wnt/Ca^2+^ pathway. Finally, given PFN1’s contrasting roles in distinct cancer types, exploring its potential as a context-dependent therapeutic target could pave the way for more personalized treatment strategies, particularly in muscle-invasive cancers where Wnt/Ca^2+^ signaling is disrupted.

### 3.2. Profilin as a Key Effector of the Non-Canonical Planar Cell Polarity (PCP) Pathway

The non-canonical Planar Cell Polarity (PCP) branch relies on a diverse set of receptors and co-receptors that integrate extracellular Wnt cues to regulate intracellular polarity signaling, cytoskeletal dynamics, and gene expression. The PCP pathway functions independently of the co-receptor LRP5/6 and is primarily mediated by Frizzled [37] and atypical cadherin-like receptors such as Celsr1 and the four-pass transmembrane protein Vangl2 [86], which assemble membrane-localized signaling complexes with Dvl, Prickle, and Inturned to transmit polarity cues across cells, though the precise ligand-binding mechanisms remain poorly defined [83,109,110].

**Figure 3:**
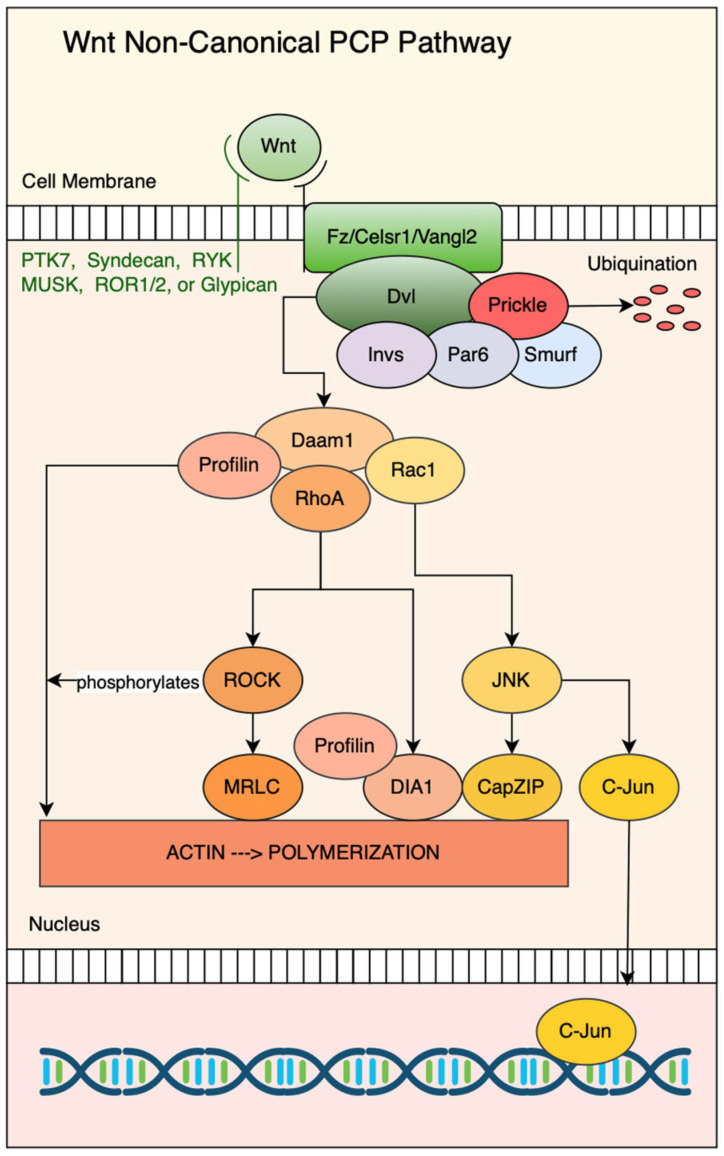
The Wnt non-canonical PCP (Planar Cell Polarity) pathway. Wnt ligands bind to Frizzled/Celsr1/Vangl2 receptors and co-receptors (PTK7, Syndecan, RYK, MuSK, ROR1/2, or Glypican) at the cell membrane, activating Dishevelled (Dvl). Downstream signaling branches through Daam1 to three central cascades: Profilin/Dia1, RhoA/ROCK/MRLC, and Rac1/JNK pathways leading to CapZIP and c-Jun. These pathways regulate actin polymerization in the cytoplasm and gene expression in the nucleus, controlling cell polarity and morphogenetic movements.

Additional PCP pathway co-receptors include protein tyrosine kinase 7 (PTK7) [111,112], muscle-specific Kinase (MuSK) [77], tyrosine kinase-like orphan receptors ROR1, ROR2 [113], the tyrosine kinase-related receptor RYK [114], and the heparan sulfate proteoglycans Syndecan and Glypican [115,116] (Figure 3). The RTK-like structural organization enables PTK receptors to recruit and organize intracellular effectors such as Dishevelled (Dvl), Src family kinases, and other polarity proteins [117,118,119,120]. PTK7 specifically interacts with Wnt5a to induce PCP signaling and modifies downstream responses by recruitment of RACK1, an adapter protein that assists in Dsh localization [121]. Membrane-associated co-receptors ROR1, ROR2, and RYK modulate Wnt ligand binding and PCP signaling output. RORs share conserved extracellular cysteine-rich domains (CRDs) similar to Fz, which allow them to bind Wnt ligands selectively and cooperatively [33].

Through these complexes, co-receptors modulate Wnt/PCP signaling in both positive and negative ways. For example, PTK7 can attenuate canonical β-catenin signaling in zebrafish embryos while promoting PCP signaling and axial elongation [122], highlighting antagonistic crosstalk between the canonical and non-canonical branches. Similarly, RORs activate the non-canonical Wnt/JNK-PCP pathway while concurrently inhibiting β-catenin-dependent signaling by competing with Frizzled receptors for Wnt ligand binding, thereby attenuating canonical signaling. ROR1/2 and RYK can bias signaling toward non-canonical pathways through preferential binding of ligands such as Wnt5a, recruiting Dvl to activate small GTPases like RhoA and Rac1, which remodel the actin cytoskeleton [123,124,125,126,127] (Figure 2). Crosstalk among these co-receptors and their interactions with cell adhesion molecules enables fine-grained modulation of Wnt signaling, balancing canonical and non-canonical outcomes. This is further regulated by secreted antagonists such as WIF-1, Cerberus, DKK, and sFRPs, which block Wnt binding to LRP5/6 or Fz [128,129]. Glypicans are membrane-bound heparan sulfate proteoglycans that also regulate the extracellular distribution and activity of Wnt ligands and modulating the signaling pathway. A recent study highlighted that glypicans exhibit an intrinsic interaction with Wnt3a in human prostate cancer cells, which is not always associated with cascade activation [116].

Upon binding to the Wnt ligand, Dvl is phosphorylated and activated. The Par6 protein interacts with Dvl, facilitating the recruitment of Smad ubiquitination regulatory factor (Smurf), which targets Prickle, an inhibitory protein for degradation [110]. The degradation of Prickle allows Dvl to associate with the Dishevelled-associated activator of morphogenesis 1 (Daam1), redirecting the signal away from canonical Wnt/β-catenin signaling. This interaction is tightly regulated by Inversin (Invs), which promotes PCP signaling by inhibiting the entry into the canonical pathway. Daam1 belongs to the formin family of actin-regulating proteins and is essential for cytoskeletal remodeling during vertebrate gastrulation [16,130,131]. Formin proteins are characterized by three functional domains, the GTPase-binding domain (GBD), the proline-rich Formin Homology 1 (FH1) domain, and the actin-nucleating Formin Homology 2 (FH2) domain. In the absence of upstream signals, formins exist in an autoinhibited conformation mediated by the Diaphanous Autoinhibitory Domain (DAD) and GBD [130,132]. Upon Dvl binding, this autoinhibition is relieved, allowing Daam1 to promote actin polymerization.

Daam1 promotes the activation of the small GTPase RhoA through its amino-terminal region by recruiting Rho-GDP and Rho-specific guanine nucleotide exchange factors (GEFs) [16]. Through its proline-rich FH1 domain, Daam1 interacts with Profilin [17]. Profilin facilitates the delivery of actin monomers to the barbed ends of growing filaments [133,134,135]. Dvl cooperatively activates both RhoA and Rac1 through distinct branches of non-canonical Wnt signaling. RhoA activation occurs via interaction with Daam1, whereas Rac1 activation is Daam1-independent [136]. Activated RhoA subsequently triggers Rho-associated kinase (ROCK), which promotes actomyosin contractility by phosphorylating the myosin regulatory light chain (MRLC), thereby modulating cytoskeletal dynamics and cell morphology [137]. Simultaneously, Rac1 activates the c-Jun N-terminal kinase (JNK) pathway, promoting transcriptional responses through c-Jun and regulating cytoskeletal dynamics via CapZ-interacting protein (CapZIP) [138]. These converging pathways driven by Daam1, MRLC, CapZIP, and Profilin coordinate the cytoskeletal rearrangements necessary for cell polarization and motility (Figure 3).

Although Profilin does not directly bind small GTPases such as Rac1 or RhoA, it connects to these pathways through intermediary scaffolding proteins. For example, Profilin2 interacts with components of the WAVE1 complex that act downstream of Rac1 to promote actin polymerization [139]. Profilin2 associates with Rho-associated coiled-coil kinase (ROCK) in a Rho-dependent manner to regulate neurite outgrowth [140]. Additionally, these interactions highlight Profilin’s involvement in Rac-mediated PCP signaling and its broader role in cytoskeletal remodeling.

Within the Planar Cell Polarity (PCP) signaling network, Profilin modulates actin filament dynamics critical for morphogenetic processes such as gastrulation and neurulation [16]. Through its actin-binding domain, Profilin catalyzes nucleotide exchange on G-actin, converting ADP-actin to ATP-actin, thereby priming monomers for incorporation into growing filaments [139,141,142]. In response to Wnt signaling, Profilin1 interacted with the FH1 domain of Daam1 and colocalizes with actin stress fibers in mammalian cells [17]. Disruption of this interaction impairs Wnt- and Daam1-induced actin reorganization, resulting in gastrulation defects such as incomplete blastopore closure in *Xenopus* embryos [17]. Furthermore, Profilin’s interaction with formins facilitates its association with microtubules and accelerates microtubule depolymerization [143]. Recent structural analyses suggest that the Profilin’s N- and C-terminal poly-L-proline (PLP) binding surface is essential for its simultaneous engagement with actin- and proline-rich effectors [144].

Another critical PCP signaling effector pathway that connects Profilin to actin and microtubule regulation is through diaphanous-related formins, particularly mDia proteins. These formins are autoinhibited until activated by RhoA [130]. Activated mDia proteins interact with Profilin-bound G-actin through their FH1 domains, facilitating actin filament elongation at the barbed end [145,146]. mDia also stabilizes the (+)-ends of microtubules, connecting Profilin to microtubule organization [147]. Notably, Profilin may bind directly to microtubules via regions distinct from its proline-rich binding interface, suggesting an additional mode of cytoskeletal integration [49].

Transgenic overexpression of Profilin1 in vascular smooth muscle cells leads to upregulation of ROCK II kinase expression, elevated actin polymerization (increased F-actin/G-actin ratio), and vascular hypertrophy in mice [148]. These findings suggest that Profilin1-driven cytoskeletal remodeling may impose mechanical stress that activates the RhoA/ROCK signaling axis, contributing to increased vascular tone and blood pressure in aged mice [148]. Platelet-specific loss of Profilin1 disrupts the organization of the adhesion-dependent circumferential actin network, leading to accelerated integrin inactivation and consequently impaired platelet function both in vitro and in vivo [149]. Profilin also cooperates with Wiskott–Aldrich syndrome protein (WASP), a nucleation-promoting factor activated by the small GTPase Cdc42. Upon activation, WASP undergoes a conformational change that exposes its Arp2/3-binding domain. Profilin may assist this process by stabilizing the active conformation of WASP or by enhancing Arp2/3-mediated actin nucleation [150,151]. These interactions collectively underscore Profilin’s role as a signaling integrator downstream of Rho family GTPases.

**Figure 4:**
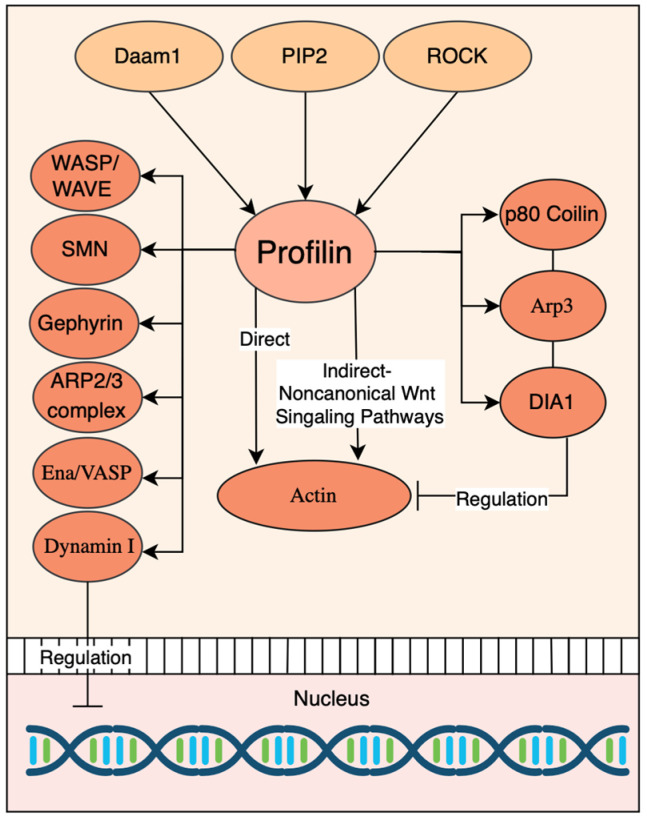
The pathway diagram demonstrates how Profilin acts as a molecular hub, integrating signals from lipid metabolism (PIP2), Rho signaling (ROCK), and other pathways to coordinate actin dynamics, membrane trafficking, and nuclear processes.

Beyond its role in cytoskeletal remodeling, Profilin contributes to centrosome positioning and mitochondrial homeostasis [152,153]. Profilin further engages in signal transduction and transcriptional regulation by interacting with a range of partners, including p80 coilin, Ena/VASP proteins [142,154], Dynamin I [139], Gephyrin [155], the Spinal muscular atrophy protein (SMN) complex [156,157]. These multifaceted roles suggest that Profilin is a central regulatory hub that links extracellular signaling cues to intracellular responses that coordinate cytoskeletal dynamics, metabolism, and gene expression. In summary, Profilin serves as a versatile molecular effector that bridges non-canonical Wnt/PCP signaling and actin cytoskeleton dynamics. Its interactions with formins, small GTPase effectors, and nucleation-promoting factors position it as an essential mediator of morphogenetic signaling during embryonic development and beyond. Figure 4 presents a network diagram illustrating the central role of Profilin in coordinating cytoskeletal dynamics, membrane trafficking, nuclear organization, and gene expression.

## 4. Role of Profilin as a Key Effector of Actin Dynamics

Profilin exerts its effects on the cytoskeleton both directly and indirectly. It directly binds to actin monomers, tubulin dimers, and microtubules, modulating actin filament turnover through interactions with cofactors such as Formins, Ena/VASP, SMN, and Exportin-6 [158]. Actin exists in two primary forms, monomeric G-actin (ATP-bound) and filamentous F-actin (ADP-bound). Actin polymerization proceeds through three phases, a lag phase, where ATP-actin monomers form unstable dimers; followed by nucleation, where a stable trimer nucleus forms; and then elongation occurs. Finally, the rapid addition of monomers to the filament’s barbed (+) end while treadmilling is established through coordinated polymerization at the barbed end and depolymerization at the pointed (−) end [159].

**Figure 5:**
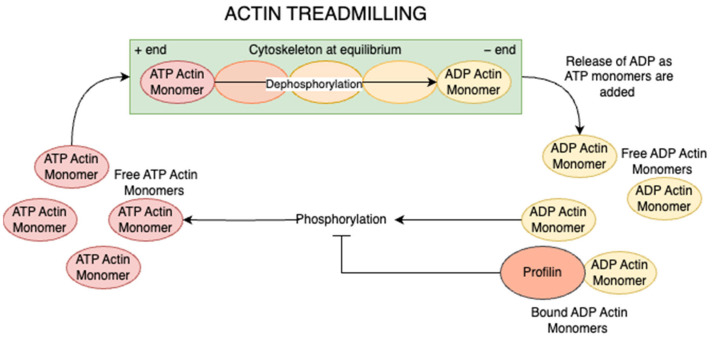
The actin treadmilling process essential for cytoskeleton dynamics. ATP-actin monomers are added to the plus end of actin filaments, while ADP-actin monomers dissociate from the minus end, maintaining cytoskeleton equilibrium. Free ADP-actin monomers are converted back to ATP-actin through phosphorylation, allowing recycling and continued polymerization. Profilin inhibits this phosphorylation by binding to ADP-actin monomers, regulating the available pool of polymerization-competent actin. This cyclical process enables controlled cytoskeletal remodeling which is necessary for cell motility and shape changes.

Profilin regulates these dynamics in a context-dependent manner. In resting cells, it sequesters ADP-actin monomers, inhibiting spontaneous filament assembly and maintaining a reserve of polymerization-ready monomers [160]. Upon activation, Profilin facilitates the exchange of ADP for ATP on actin monomers. It recruits these ATP-actin-Profilin complexes to the PLP domains of Formin and Ena/VASP proteins, promoting filament elongation (Figure 5) [158]. Profilin also coordinates with other regulators, such as thymosin β4, Arp2/3, gelsolin, and cofilin, to fine-tune actin architecture [161]. Additionally, Profilin is involved in N-terminal acetylation of actin, a modification that modulates interactions with actin-binding proteins [162]. Profilin facilitates the N-terminal acetylation of actin by serving as the binding platform for the acetyltransferase NAA80 on the Profilin–actin complex, a modification essential for actin’s interaction with actin-binding proteins [163].

## 5. Expression and Function of Profilin Genes Across Species

Profilin is a critical regulator of actin dynamics and has additional roles that extend beyond the cytoskeleton. The existence of multiple Profilin isoforms, each with tightly regulated expression in specific tissues across diverse species, underscores the need for precise spatial and temporal control of Profilin activity. This isoform diversity enables fine-tuned regulation of cytoskeletal remodeling, cellular signaling, and development. Below, we summarize the expression patterns and functional roles of Profilin isoforms across representative model organisms, highlighting both conserved and species-specific features.

### 5.1. Human Profilins

Profilin1 (PFN1): Profilin1 is ubiquitously expressed across a wide range of human tissues, including the placenta, lung, liver, skeletal muscle, and pancreas [164]. Functionally, PFN1 regulates endothelial cell migration and proliferation. RNA interference studies in human umbilical vein endothelial cells (HUVECs) demonstrated that PFN1 knockdown impairs actin filament formation, reduces focal adhesion assembly, and disrupts cell–cell adhesion dynamics. These alterations result in diminished cell motility, membrane protrusion, and directional persistence without significantly affecting short-term survival [165]. PFN1 also acts as a negative regulator of cytotoxic T-lymphocyte (CTL)-mediated cytotoxicity [166] and contributes to mitochondrial homeostasis. In the context of amyotrophic lateral sclerosis (ALS), PFN1 mutations impair autophagy and mitochondrial integrity [167].

Profilin2 (PFN2): Two splice variants, PFN2a and PFN2b, have been identified for human PFN2 [168]. PFN2 is broadly expressed across tissues but generally exhibits an inverse expression pattern to PFN1, with this trend most evident in the brain and skeletal muscle [164]. Elevated PFN2 levels in serum and exosomes from myocardial infarction patients suggest a role in post-injury angiogenesis [169]. Functionally, PFN2 suppresses epithelial–mesenchymal transition (EMT) and metastasis in colorectal cancer by modulating cytoskeletal reorganization and myosin light-chain phosphorylation. Reduced PFN2 expression correlates with enhanced invasiveness and metastasis, whereas its overexpression dampens these features [170].

Profilin3 (PFN3): PFN3 is predominantly expressed in the kidneys and testes [171,172]. It lacks several conserved actin-binding residues, limiting its role in actin regulation [173]. In the testis, PFN3 appears during late spermatogenesis and likely contributes to cytoskeletal remodeling during sperm maturation [172].

Profilin4 (PFN4): PFN4 is expressed exclusively in the testes. It does not bind to actin or proline-rich ligands. Instead, it selectively interacts with specific phosphoinositides [173]. During spermiogenesis, PFN3 and PFN4 sequentially localize to the acroplaxome and manchette [174]. PFN4 expression serves as a molecular diagnostic marker in human testicular biopsies, supporting its use in conjunction with conventional histopathology [172].

### 5.2. Chicken Profilins

In embryonic chicken fibroblasts, knockdown studies revealed a non-redundant role for PFN2a but not PFN1 in regulating cell adhesion and motility [175]. During development, both PFN1 and PFN2a are co-expressed, yet PFN1 expression diminishes in adult heart, liver, and kidney tissues, whereas PFN2a remains consistently expressed [175], suggesting isoform-specific adaptation to tissue needs.

### 5.3. Mouse Profilins

Profilin1: Whole-body PFN1 knockout results in embryonic lethality, highlighting its essential role in early development. Notably, PFN2a cannot compensate for the loss of PFN1, reinforcing their distinct roles [176].

Profilin2: Predominantly expressed in the brain, PFN2a regulates actin polymerization at synapses via the WAVE complex. PFN2 knockout disrupts synaptic actin remodeling, leading to increased neurotransmitter release and striatal activity, which is associated with novelty-seeking behavior [177]. PFN2a and PFN2b differ in their C-terminal sequences and interaction networks [178]. Developmentally, PFN2a mRNA appears at embryonic day (E) 9.5-E10, with protein levels rising postnatally [168].

Profilin3 (PFN3): PFN3 is enriched in the testes and kidneys, similar to its human counterpart. PFN3 is upregulated in diabetic nephropathy and polycystic kidney disease [171]. Male PFN3-deficient mice display globozoospermia due to defective manchette formation and malformed sperm heads [179].

Profilin4: Restricted to the testes, PFN4 is essential for manchette formation and acrosome biogenesis. Male PFN4-deficient mice are infertile, while females exhibit normal fertility [180].

### 5.4. Bovine Profilins

Profilin is present throughout early bovine embryonic stages. Its inhibition leads to cytoskeletal disruption, cleavage failure, and arrested embryo development [156]. Tissue-specific expression reveals that PFN1 predominates in the spleen, whereas PFN2 is abundant in the brain. Purified bovine PFN2 binds actin and poly (L-proline) with higher affinity than PFN1, particularly at acidic pH, due to C-terminal amino acid differences [181].

### 5.5. Rat Profilins

Rat PFN1 shares about 49% sequence identity with *Xenopus* PFN1. In the testes, PFN3 and PFN4 are expressed in spermatogenic cells with distinct temporal localization patterns. Initially present in the acroplaxome, both isoforms later localize to the manchette, where they likely regulate cytoskeletal remodeling during sperm head formation [174].

### 5.6. Frog (Xenopus) Profilins

Profilin1: In *Xenopus*, PFN1 is required for blastopore closure during gastrulation but is not involved in convergent extension or neural fold closure [17].

Profilin2: *Xenopus* Profilin2 plays a more critical role than PFN1 in morphogenesis. Loss- or gain-of-function studies demonstrate that altered PFN2 levels result in gastrulation defects, including shortened anterior–posterior axis, curved body, and open neural tube [19]. Notably, Profilin3 has not been identified in *Xenopus*, while Profilin4 retains a conserved gene structure shared with other vertebrates [182].

### 5.7. Zebrafish Profilins

Zebrafish express multiple Profilin isoforms, including zpfn1, zpfn2a, zpfn2b, zpfn3, and zpfn4. Functional analysis shows that zpfn1 is critical during gastrulation. Morpholino knockdown of zpfn1 causes about 28% of embryos to exhibit epiboly and convergent extension defects, whereas zpfn2 knockdown results in minimal abnormalities (about 1.6%) [18]. Co-injection of zpfn1 and zDia2 morpholinos exacerbates these defects, whereas zpfn2 exhibits no synergistic interaction. However, the specific zpfn2 isoform used was not identified, and comprehensive temporal–spatial expression data remain lacking. A comparative analysis of Profilin gene expression and functions across metazoan species is presented in Table 1.

## 6. Dysregulation of Profilin and Its Implications in Disease

Given its pivotal role in cytoskeletal dynamics and intracellular signaling, it is not surprising that dysregulation of Profilin contributes to a diverse array of pathological conditions. Aberrant Profilin expression or function has been implicated in neurological disorders, cardiovascular disease, cancer progression, and metabolic complications [183]. Some studies have connected PFN1 to a degenerative bone condition called Paget Disease [184,185].

### 6.1. Neurological Disorders

Profilin dysfunction is notably linked to neurodegenerative diseases. Our review underscores the importance of Profilin’s roles in a subset of prominent neurological pathologies, including amyotrophic lateral sclerosis (ALS), Fragile X syndrome (FXS), Huntington’s disease (HD), and spinal muscular atrophy (SMA) [186].

#### 6.1.1. Amyotrophic Lateral Sclerosis (ALS)

Exome sequencing has identified eight PFN1 mutations (C71G, G118V, M114T, E117G, T109M, R136W, A20T, Q139L) in familial and sporadic ALS cases [187,188,189,190,191]. These mutants recapitulate ALS pathology in rodent models, inducing cytoskeletal defects such as reduced F-/G-actin ratios, dendritic shortening, and axonal degeneration [192,193,194]. Although initial studies suggested disrupted G-actin binding due to proximity of mutations to the actin-binding site [187], later biochemical assays showed that G-actin affinity remains largely intact in most PFN1 mutants [195,196]. Instead, protein instability and aggregation emerge as key mechanisms. Aggregation-prone mutants like C71G, G118V, M114T, and A20T form insoluble, ubiquitinated inclusions in neurons and cultured cells, unlike wildtype PFN1 [187,190]. Biochemical and structural studies confirm that these mutations destabilize protein folding, exposing hydrophobic cores that promote aggregation [195,197,198]. C71G, in particular, exhibits the highest instability due to a deep internal cavity and is rapidly targeted for proteasomal degradation [199]. In contrast, E117G and Q139L show minimal aggregation and mild phenotypes, with E117G possibly representing a benign variant [190,195]. These findings support the view that mutation-driven conformational destabilization and cytosolic aggregation of PFN1 are central to ALS pathogenesis.

#### 6.1.2. Fragile X Syndrome (FXS)

FXS is an X-linked neurodevelopmental disorder marked by intellectual disability, hyperactivity, repetitive behaviors, and autism-like features. It results from mutations in the FMR1 gene affecting the Fragile X Mental Retardation Protein (FMRP), which regulates mRNA localization, stability, and translation [200]. In Drosophila, FMRP binds Profilin (chickadee) mRNA, with FMRP mutants showing elevated Profilin levels that disrupt axonal pruning [201,202]. Conversely, in mice, PFN1 but not PFN2a levels are reduced in FMR1 mutants. PFN1 overexpression rescues cortical development defects, and PFN1 loss impairs dendritic spine formation, mimicking FMRP-deficient phenotypes [203,204,205]. Evidence for direct FMRP-PFN1 mRNA binding is mixed, and it remains unclear whether PFN1 dysregulation occurs in human FXS patients.

#### 6.1.3. Huntington’s Disease (HD)

HD is an autosomal dominant neurodegenerative disease caused by expanded CAG repeats in huntingtin gene, leading to toxic polyglutamine (polyQ) huntingtin protein aggregation, particularly in the striatum [206,207]. Huntingtin contains two proline-rich domains (PRDs) that bind PFN1 and PFN2, reducing aggregation and toxicity by stabilizing soluble huntingtin states [208,209,210]. PFN1 binding prevents formation of aggregation-prone tetramers and stabilizes monomers/dimers [210]. However, this protective effect is lost once large aggregates form. ROCK-dependent phosphorylation of PFN1 at Ser138 disrupts huntingtin binding [208], and ROCK inhibition reduces aggregation and toxicity in cell and animal models [211,212]. Notably, reduced Profilin levels correlate with HD progression in patients [213].

#### 6.1.4. Spinal Muscular Atrophy (SMA)

Additionally, Profilin dysregulation has been associated with spinal muscular atrophy, further emphasizing its critical role in neuronal survival and cytoskeletal regulation [186]. SMA is caused by mutations or deletions in *SMN1*, leading to motor neuron degeneration and muscle atrophy. SMN interacts with both PFN1 and PFN2a, binding more strongly to PFN2a via its C-terminal poly-proline motifs [214]. In PC12 cells and motoneurons, PFN1/2a co-localize with SMN [214,215]. SMN modulates PFN2a’s role in actin polymerization and regulates its expression. In mammals, SMN depletion alters PFN2a levels and its phosphorylation by ROCK, leading to cytoskeletal changes such as stress-related actin rods and impaired neurite outgrowth [186,216].

### 6.2. Cardiovascular and Metabolic Disease

In the vascular system, Profilin overexpression contributes to endothelial dysfunction and vascular pathology. Profilin1 was originally identified as a binding partner for a diabetic aorta-specific phage and was found to be elevated in the aortic endothelium of diabetic patients and streptozotocin-induced diabetic rats [183]. Profilin was also enriched in atherosclerotic plaques, suggesting its contribution to vascular inflammation and remodeling. These findings position Profilin as a mediator of endothelial dysfunction in the pathogenesis of diabetes and atherosclerosis. Profilin overexpression has also been associated with vascular hypertrophy and hypertension [148], and elevated PFN2 levels were recently reported in the serum and exosomes of myocardial infarction patients [169], implicating Profilin in acute cardiovascular events.

### 6.3. Cancer Biology

Profilin exhibits context-dependent roles in cancer. In various cancers, PFN1 and PFN2 have been shown to exhibit dual functions acting either as tumor suppressors or displaying oncogenic potential depending on the specific cancer cell type involved (for a comprehensive review, see [217]). In triple-negative breast cancer, elevated levels of PFN1 correlate with tumor progression and metastasis. It can also enhance actin polymerization and cytoskeletal remodeling, processes that facilitate cell motility and invasiveness [218]. While PFN1 overexpression may suppress tumor growth in certain settings by promoting the accumulation of the cell cycle inhibitor p27, it can also enhance actin polymerization and cytoskeletal remodeling, processes that facilitate cell motility and invasiveness. In small-cell lung cancer (SCLC), PFN2 is significantly upregulated in tumor tissues relative to normal lung, where it supports tumor angiogenesis and metastatic dissemination [219]. These studies highlight the complex, sometimes paradoxical, roles of Profilin isoforms in tumor biology, functioning either as tumor suppressors or oncogenic facilitators depending on cellular context and isoform expression.

### 6.4. Clinical Biomarker Potential

Emerging evidence suggests that Profilin may serve as a diagnostic or prognostic biomarker in specific pathological conditions. For example, elevated Profilin levels have been proposed as potential biomarkers for preeclampsia, reflecting its role in endothelial stress and placental dysfunction [220]. Similarly, increased Profilin expression has been detected in patients with pulmonary thromboembolism, indicating possible diagnostic utility in thrombotic disorders [219]. Excessive PFN1 release has been shown to contribute to endothelial dysfunction by promoting vascular remodeling, including increased vessel diameter, wall thickness, collagen deposition, and low-density lipoprotein (LDL) accumulation [221]. Elevated PFN1 levels have also been reported in hypertensive patients, likely due to enhanced vascular thickening and angiogenesis [222]. In acute myocardial infarction, PFN1 levels rise in parallel with platelet accumulation within thrombi, underscoring its potential relevance as a thrombus-associated biomarker.

## 7. Concluding Remarks

Advancements in Wnt signaling research have shed light on the multifaceted roles of Profilin, particularly in cytoskeletal regulation, cell signaling, and disease pathology. While Profilin’s classical function in actin dynamics is well established, it is increasingly recognized as a critical mediator of signal transduction, gene expression, metabolism, and mitochondrial function. Profilin’s ability to integrate signaling events with cytoskeletal remodeling underscores its central role in processes such as cell motility, division, and morphology.

Significant progress has been made in characterizing PFN1 and PFN2a, particularly in mammals and frogs; however, the physiological roles of less-studied isoforms PFN2b, PFN3, and PFN4 remain poorly understood. Their functions in non-neuronal and reproductive tissues, as well as across vertebrate species, merit detailed investigation. Comparative studies using zebrafish, *Xenopus*, and avian models will be invaluable in deciphering isoform-specific roles and their evolutionary conservation.

Emerging evidence places Profilin at the intersection of non-canonical Wnt signaling pathways, notably the Planar Cell Polarity (PCP) and Wnt/Ca^2+^ branches. However, the mechanistic interplay between these pathways in development and disease contexts, particularly in cancer, neurodegeneration, and vascular disorders, remains largely unexplored. Elucidating how Profilin modulates crosstalk between these signaling cascades may identify novel regulatory mechanisms and therapeutic targets.

Given the correlation between aberrant Profilin expression and disease states, future efforts should prioritize the development of isoform-specific antibodies and molecular probes to assess the diagnostic and prognostic value of Profilin in clinical settings. Investigating post-translational modifications, including phosphorylation and acetylation, may reveal additional layers of isoform-specific regulation. Moreover, understanding how Profilin localizes to subcellular structures such as centrosomes and mitochondria could uncover new functional domains or interacting partners with roles in embryogenesis and cell fate determination. Ultimately, dissecting the biological roles of individual Profilin isoforms in vivo will deepen our understanding of cytoskeletal regulation and may unlock new avenues for treating Profilin-associated pathologies.

## Figures and Tables

**Table 1:** Comparative table of Profilin isoform expression and functions across species.

Organism	Gene(s)	Expression Pattern	Known Functions	References
Human	PFN1	Ubiquitous	Cytoskeleton organization, endothelial migration, mitochondrial homeostasis, ALS, CTL regulation	[164]
	PFN2a/b	Broad; enriched in brain	Tumor suppression, angiogenesis post-MI	[168,169,170]
	PFN3	Kidney, testis	Spermatogenesis; lacks actin-binding	[171,172,173]
	PFN4	Testis-specific	Sperm maturation, PIP-binding, diagnostic marker	[172,174]
Mouse	PFN1	Ubiquitous	Embryonic survival,	[176]
	PFN2a/b	Neuronal	Synaptic plasticity, actin regulation, behavior	[177,178]
	PFN3	Kidney, testis	Manchette development, sperm morphology	[179]
	PFN4	Testis-specific	Manchette and acrosome formation, male fertility	[180]
Chicken	PFN1	Declines post-embryo	Less critical for adhesion/motility	[175]
	PFN2a	Embryonic and adult	Cell adhesion and locomotion	[175]
Bovine	PFN1, PFN2	PFN1: spleen, PFN2: brain; early embryo	Required for cleavage and blastocyst formation; PFN2 strongly inhibits actin polymerization	[156,168]
Rat	PFN1, PFN3, PFN4	Testis (PFN3, PFN4)	Sperm development (acroplaxome, manchette)	[174]
*Xenopus*	PFN1	Gastrulation stage	Blastopore closure	[17]
	PFN2	Only isoform	Neural tube closure, convergent extension	[19]
	PFN3	Not identified	-	-
	PFN4	Conserved structure	Presumed role in spermatogenesis	[182]
Zebrafish	PFN1 (zpfn1)	Early development	Gastrulation, epiboly, zDia2 interaction	[18]
	PFN2 (zpfn2)	Weak phenotype	Minor role, no synergy with zDia2	[18]
	PFN3, PFN4, PFN2a/b	Predicted, uncharacterized	Expression and function unknown	-
